# A Review of Modern Control Strategies for Clinical Evaluation of Propofol Anesthesia Administration Employing Hypnosis Level Regulation

**DOI:** 10.1155/2017/7432310

**Published:** 2017-03-30

**Authors:** Muhammad Ilyas, Muhammad Fasih Uddin Butt, Muhammad Bilal, Khalid Mahmood, Ali Khaqan, Raja Ali Riaz

**Affiliations:** ^1^Department of Electrical Engineering, COMSATS Institute of Information Technology, Islamabad 45550, Pakistan; ^2^Department of Electrical Engineering, Iqra National University, Peshawar 25000, Pakistan

## Abstract

Regulating the depth of hypnosis during surgery is one of the major objectives of an anesthesia infusion system. Continuous administration of Propofol infusion during surgical procedures is essential but it unduly increases the load of an anesthetist working in a multitasking scenario in the operation theatre. Manual and target controlled infusion systems are not appropriate to handle instabilities like blood pressure and heart rate changes arising due to interpatient and intrapatient variability. Patient safety, large interindividual variability, and less postoperative effects are the main factors motivating automation in anesthesia administration. The idea of automated system for Propofol infusion excites control engineers to come up with more sophisticated systems that can handle optimum delivery of anesthetic drugs during surgery and avoid postoperative effects. A linear control technique is applied initially using three compartmental pharmacokinetic and pharmacodynamic models. Later on, sliding mode control and model predicative control achieve considerable results with nonlinear sigmoid model. Chattering and uncertainties are further improved by employing adaptive fuzzy control and *H*_*∞*_ control. The proposed sliding mode control scheme can easily handle the nonlinearities and achieve an optimum hypnosis level as compared to linear control schemes, hence preventing mishaps such as underdosing and overdosing of anesthesia.

## 1. Introduction

General anesthesia is a broad term including the use of drugs to induce and maintain the following three states during surgery: hypnosis (depth of unconsciousness), analgesia (absence of pain), and areflexia (lack of movement). Most surgeries use multiple anesthetic drugs in order to achieve all these states. Before the advent of anesthesia, surgical operations needed fast execution. Different techniques such as cold and hot treatment are used to provide slight relief from pain. The discovery of inhaled gases which stimulates patients to the state of unconsciousness made invasive surgeries possible [1]. The first anesthesia process was performed by Crawford Williamson using diethyl ether. By inhaling ether, he realized that it has the ability to provide insensitiveness against pain. The term anesthesia was later proposed by Morton, which means lack of esthesia, that is, sense. Due to increasing complexities of administration and management of anesthesia, it was clear that it required expertise and specialties of anesthesiologists. It is estimated that about 150 people die every year due to complications in anesthesia in United State of America [2].

The primary aim of anesthetics is to deliver painless feeling during execution of surgery in patients. Evolution in scientific and surgical procedures has completely altered clinical surgery through the application of modern medicine. Such incredible breakthroughs are possible through research outcomes in modern anesthesia [3]. Propofol is a hypnotic agent used in general surgery. Its importance lies in its fast metabolism and because it has no side effects on the patients [4]. Inappropriate anesthetic delivery can cause severe consequences during and after a surgery. If not enough anesthetic is delivered, the patient can remain conscious during surgery, which causes trauma, anxiety, and vomiting. Too much infusion of anesthetic drugs can cause a patient to stop breathing and can result in a cardiovascular collapse. Both of these conditions describing underdosing and overdosing are unaffordable and unacceptable throughout surgery in terms of health and safety [1, 2]. Delivery of anesthetic agent during surgery is traditionally manually controlled by anesthetists. The first step involves the selection of an appropriate drug and dosage level according to patient weight and age and the type of surgical operation. The next step deals with the performance of medical equipment in the operating room to monitor the vital signs regarding patient safety and warns the anesthesiologist in unexpected circumstances. The last step focuses on the experience and knowledge of anesthesiologist to handle the unpredictable conditions during surgical procedure. Hence, the human error of anesthesiologist during a surgical activity related to excessive or unbalanced amount of drug can be dangerous or even life-threatening to the patient [5]. Therefore, modern clinical practices need well equipped operation theatres where unpredictable measures can be safely handled. For this reason, automated closed loop control of anesthesia needs to be studied to establish its significance in control engineering community as well as biomedical field. Hence, a lot of work remains to be done in order to demonstrate the safety and efficacy of such systems. The closed loop controller design is based on a set of models which describes the interpatient variability in response to Propofol infusion [6, 7]. This model consists of pharmacokinetics (PK) describing how the drug is metabolized by the body and pharmacodynamics (PD) showing the drug's effect on the depth of hypnosis (DOH). The drug distribution in the body depends on the metabolism rate within different organs including muscles, bones, and fats [8]. Anesthesia can be conveniently classified into three functional states, each being associated with different drugs [9]. The main functional component of anesthesia is “hypnosis.” Its purpose is to take the patient to a state which prevents the perception and recall of noxious stimuli. Although an acceptable hypnotic state prevents the patient from perceiving or evoking noxious stimuli, the cortical activity of patient is monitored through Bispectral Index Scale (BIS). Standard range of hypnosis level on BIS is 40 to 60 for general surgery. Propofol is the hypnotic drug which is also called inducer in medical terminology [9–11]. The second ingredient of anesthesia is “analgesia” which means lack of pain. Before an inducer analgesic is given to the patient, a small amount of analgesic is continuously administrated to the patient during a surgical procedure. After completion of the surgical procedure, analgesics are given to the patient in intervals. The final objective of anesthesia is “immobility.” It is the third state of anesthesia. Certain muscles, mostly abdominal ones, show reflex activity. This activity is naturally not blocked in an acceptable hypnotic and analgesic state, thus provoking the use of paralyzing drugs which can result in a neuromuscular blockade. The resulting immobility state of anesthesia is completely decoupled from hypnosis and analgesia, which permits separate treatment from a control engineering perspective [12]. During manual delivery of anesthesia, several clinical problems including underdosing and overdosing are encountered. Underdosing of anesthetics during surgical procedure causes vomiting and may result in making the patient aware and anxious, while overdosing leads to cardiovascular collapse. These circumstances are undesirable during surgery [13]. These complications in anesthesia administration create a big room for control engineering community to introduce automation in anesthesia. This paper proposes a complete review of clinical evaluation of Propofol anesthesia administration employing modern control strategies. The rest of the paper is organized as follows.  Section 2 describes the work flow of anesthesia during surgical procedures.  Section 3 explains the mathematical model and its analysis with different bioparameters, while Section 4 describes different control schemes applied in silico and real patients including their performance analysis.  Section 5 describes assessment and analysis of closed loop anesthesia based on simulation results. Finally, Section 6 presents our conclusion.

## 2. Anesthesia Administration in Manual Surgical Procedures

In intensive care unit (ICU), anesthesiologists use hypnotic as well as analgesic to prevent the awareness of patient to the pain and intensify the body stress to injury. There are three main phases of anesthesia: induction, maintenance, and emergence phases [14, 15].

### 2.1. Temporal Phases of Anesthesia Administration

**Figure 1:**
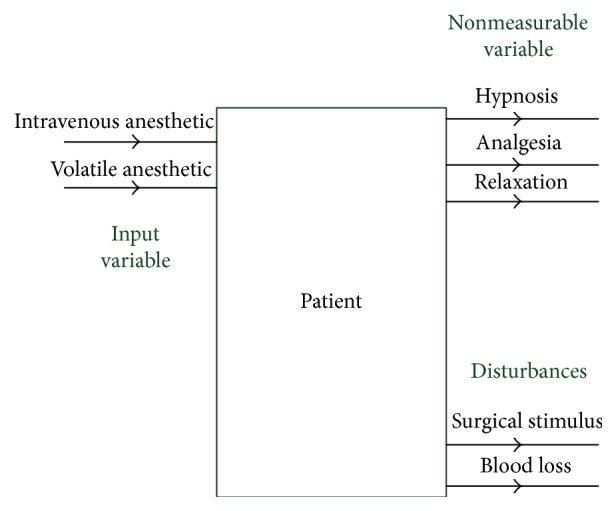
Input and output description of the anesthetic process [8].

The induction phase of anesthesia (or simply induction) is a transient phase during which the patient undergoes transition from being awake to an adequate anesthetic state. The duration of induction phase lasts from 20 to 30 seconds, but anesthetic agent may take some time to attain the desired hypnosis level [16]. This functional definition was first proposed by Pry-Roberts in 1989 observing hypnosis and amnesia [17]. Immobility is initiated by spinal reflexes suppression termed as areflexia. Hypnosis changes the cortical activity of the brain leading to desired level of unconsciousness. Analgesic acts as a painkiller administered continuously during surgical procedure [17]. Before the initiation of surgical procedure, the anesthesiologist must know the weight, age, and gender of the patient [18, 19]. Attaining the desired hypnosis level marks the transition from induction phase to maintenance phase, in which the surgical procedure is performed. During skin incision, the depth of hypnosis level may move towards the awareness state. Controller robustness handles this uncertainty while maintaining the required depth of hypnosis (DOH) level. Different patients have different DOH responses to a drug. Robust controller also handles the interpatient variability [10, 16]. Emergence phase is started when surgical procedure is near completion or at the time of skin closure. This phase is not important from control perspective; hence, the control signal is disabled during this phase and DOH level moves towards 100 indicating completely awake state [19]. The whole process is presented in Figure 1, which represents the input and output description of the anesthetic process [8].

### 2.2. Intraoperative Awareness of the Patient

Intraoperative awareness is the consciousness of patient during surgical procedure. Sometimes surgical procedure gets prolonged due to medical complexities. Awareness of pain causes psychological consequences during surgical procedure. Awareness is more common when there is presence of pain. Surveys have been carried out for observing intraoperative awareness whose range was noted to be between 0.2% and 1.6% [20, 21]. The development of monitoring tools to judge whether the patients are properly hypnotized or not has been the focus of many researchers. Based on the review of 4183 individuals in USA in 1961, it was estimated that intraoperative awareness in women is higher as compared to men [22].

### 2.3. Anesthetic Drugs

This section discusses some characteristics of drugs used to provide an adequate hypnotic state during surgical procedures. Propofol is an intravenously administrated drug which is used to control hypnotic state of the patient during surgery. There are various volatile drugs which are inhaled and their action is not purely hypnotic; instead they act as painkillers. Nitrous oxide is an example of a volatile drug. Propofol has the ability to metabolize faster and be absorbed within the body rapidly. Propofol is not accumulated in the body tissues and is less harmful to kidneys. Due to all these features, Propofol has been used since 1990 [9, 16]. It is not possible for the anesthesiologists to measure the drug's effect and the depth of anesthesia directly. Hence, they deduct the depth of hypnosis level from the clinical conditions of patient like blood pressure, pupil movement, and so forth. In modern ICU and operation theatre, there is also BIS monitor to measure electroencephalograph (EEG) of the patient. EEG of patient changes with infusion of hypnotic agent. EEG waveform represents the cortical activity of the patient [23].  Table 1 presents different ingredients of anesthetics including Propofol and Remifentanil. Their dosage levels are different for different surgical procedures [24].

### 2.4. Bispectral Index Scale

**Figure 2:**
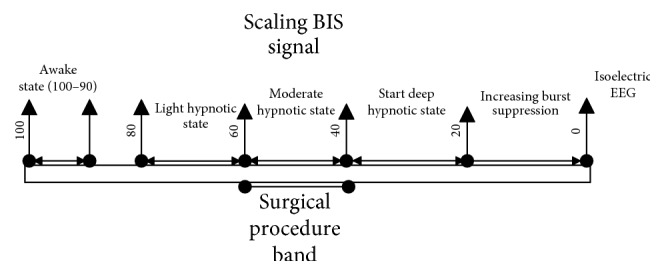
BIS for general surgery.

The impact of anesthetics on electroencephalogram has been known to neurophysiologists since 1940. They observed that anesthetized men have slower wave with higher amplitude. They used different algorithms to extract the information from EEG [25]. The EEG waveform and the clinical effect are measured using BIS. BIS markings range from 0 (no action in cerebral cortex) to 100 (fully conscious state), as shown in Figure 2. Desired region for general surgery is 40 to 60 [26].

### 2.5. Pharmacokinetics

PK describes how the drug is absorbed by the body. The basic terms of pharmacokinetics are clearance and volume [27]. Absorption and distribution of the drug depend on metabolism level of the body. Young patients can easily dissolve the drug and will be hypnotized more rapidly. Infusion drug is metabolized within human body in an exponential fashion [28].

### 2.6. Pharmacodynamics

PD describes how the drug affects the DOH. This standard PK/PD model is a compartmental model. The effect of drug is measured at the brain side showing the level of unconsciousness. Inducing hypnotic agent in human reduces the cortical activity of patient. Anesthesiologists also focus on the clinical signs during a surgical procedure [29].

## 3. Compartmental Model of the Patient

**Figure 3:**
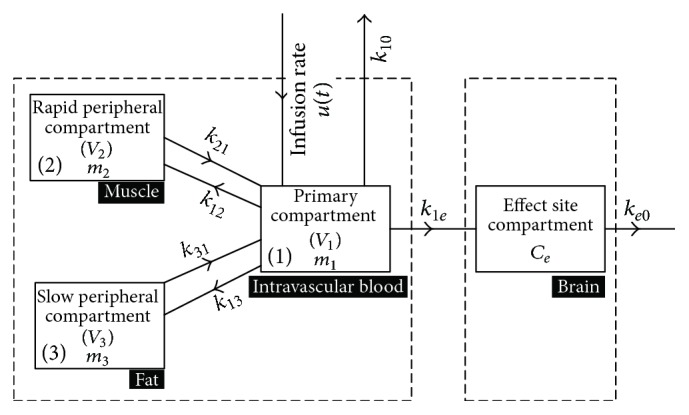
Block diagram of PK and PD models.

The dynamics of the hypnotic drug is categorized in its PK and PD parameters. The PK parameter is used to govern the behavior of the infused drug in the body over time including its distribution, metabolism, absorption, and clearance, while the PD parameter represents the drug's concentration in the blood and the corresponding impact caused at the effect site [6]. On the basis of blood flow in different organs, medical literature divides human body into various compartments. Compartmental model represents a basic kinetic approach to describe drug absorption, distribution, and elimination [1]. This model, which relates plasma drug levels to PD parameters, is intensively used in various biomedical and biotechnical applications because of its inherent flexibility and simplicity. The integrated PK/PD structure follows compartmental model. In the present study, a three-compartment PK model with an additional effect compartment has been adopted, owing to its sufficient precision and computational efficiency [30–32]. Centred on a primary compartment (intravascular blood) with volume *V*_1_, a rapid peripheral compartment (muscle) and a slow peripheral compartment (fat), with volumes *V*_2_ and *V*_3_, respectively, are connected to the primary compartment. Thus, distribution and elimination of the drug between primary and peripheral compartments takes place with weighted rate constants (*k*_12_, *k*_21_, *k*_13_, *k*_31_) as depicted in Figure 3. At any time, the change in concentration of the drug in primary compartment is related to the drug moving to and from the rapid and slow peripheral compartments. The induction and clearance of the drug take place through the primary compartment. The drug is eliminated from this compartment in an exponential fashion [33]. At the effect site (brain), the concentration of the drug is measured through the cortical activity in the brain, which is calculated from the modified form of EEG signal [5]. The extracted information can then be mapped to DOH in order to analyze patient's suitability for surgical procedures.

*k*_*ji*_ are the intercompartmental constants representing the amount of drug flow from one compartment to the other. *u*(*t*) is input hypnotic agent into the primary compartment (intravascular blood) [34]. To derive the PK model, state equations corresponding to the three compartments can be written as

(1) m1˙t=−k10m1t−k12m1t−k13m1t+k21m2t+k31m3t+ut,

(2) m2˙t=k12m1t−k21m2t,

(3) m3˙t=k13m1t−k31m3t.

Laplace transform of (1)–(3) yields the following:

(4) sM1s=−k10+k12+k13M1s+k21M2s+k31M3s+Us,

(5) sM2s=k12M1s−k21M2s,

(6) sM3s=k13M1s−k31M3s.

Solving (4)–(6), the input-output relationship can be written as

(7) $$\begin{array}{c} D_{p}\left(\;s\;\right)=\frac{M_{1}\left(s\right)}{U\left(s\right)}=\frac{\left(s^{2}+s\left(k_{21}+k_{31}\right)+k_{21}k_{31}\right)}{\left(s^{3}+s^{2}\left(k_{10}+k_{12}+k_{21}+k_{13}+k_{31}\right)+s\left(k_{10}k_{21}+k_{10}k_{31}+k_{12}k_{31}+k_{13}k_{21}+k_{31}k_{21}\right)+k_{10}k_{21}k_{31}\right)}, \end{array}$$

where *D*_*p*_(*s*) is the rate of drug absorption/metabolism within the body defined as disposition rate. Rewriting (7), the general form of PK model is obtained as

(8) $$\begin{array}{c} D_{p}\left(\;s\;\right)=\frac{M_{1}\left(s\right)}{U\left(s\right)}=\frac{b_{2}s^{2}+b_{1}s+b_{0}}{a_{3}s^{3}+a_{2}s^{2}+a_{1}s+a_{0}}, \end{array}$$

where *b*_2_ = 1, *b*_1_ = *k*_21_ + *k*_31_, *b*_0_ = *k*_21_*k*_31_, *a*_3_ = 1,  *a*_2_ = (*k*_10_ + *k*_12_ + *k*_21_ + *k*_13_ + *k*_31_), *a*_1_ = (*k*_10_*k*_21_ + *k*_10_*k*_31_ + *k*_12_*k*_31_ + *k*_13_*k*_21_ + *k*_31_*k*_21_), and *a*_0_ = *k*_10_*k*_21_*k*_31_.

The PD model indicating level of consciousness relates concentration of the drug in plasma to the effect site concentration and can be derived based on the state equation; that is,

(9) $$\begin{array}{c} {\dot{m}}_{e}\left(\;t\;\right)=k_{1e}m_{1}\left(\;t\;\right)-k_{e0}m_{e}\left(\;t\;\right). \end{array}$$

Applying Laplace transform on (9), we get

(10) $$\begin{array}{c} sM_{e}\left(\;s\;\right)=k_{1e}M_{1}\left(\;s\;\right)-k_{e0}M_{e}\left(\;s\;\right). \end{array}$$

Considering that *k*_1*e*_ and *k*_*e*0_ are equal because of the negligible volume of the effect site compartment, the disposition rate at the effect side is given by

(11) $$\begin{array}{c} D_{e}\left(\;s\;\right)=\frac{M_{e}\left(s\right)}{M_{1}\left(s\right)}=\frac{k_{e0}}{\left(s+k_{e0}\right)}. \end{array}$$

Based on the cascaded nature of PK and PD models, the overall patient model can finally be written as

(12) $$\begin{array}{c} H_{p}\left(\;s\;\right)=\frac{k_{e0}}{\left(s+k_{e0}\right)}*\frac{b_{2}s^{2}+b_{1}s+b_{0}}{a_{3}s^{3}+a_{2}s^{2}+a_{1}s+a_{0}}. \end{array}$$

BIS is related with anesthetic effect site concentration *C*_*e*_(*t*) through nonlinear sigmoid model; that is,

(13) $$\begin{array}{c} BIS\left(\;t\;\right)=E_{0}-E_{max}*\frac{{C_{e}\left(t\right)}^{\gamma }}{\left({C_{e}\left(t\right)}^{\gamma }+C_{50}^{\gamma }\right)}, \end{array}$$

where *C*_*e*_(*t*) can be computed by integrating the following equation:

(14) $$\begin{array}{c} {\dot{C}}_{e}=-0.1068m_{1}+0.456C_{e}. \end{array}$$

Table 2 presents clinical parameters expressed in compartmental model of the patient, their units, and nomenclature [34].

## 4. Propofol Infusion with Linear and Nonlinear Control Schemes

**Figure 4:**
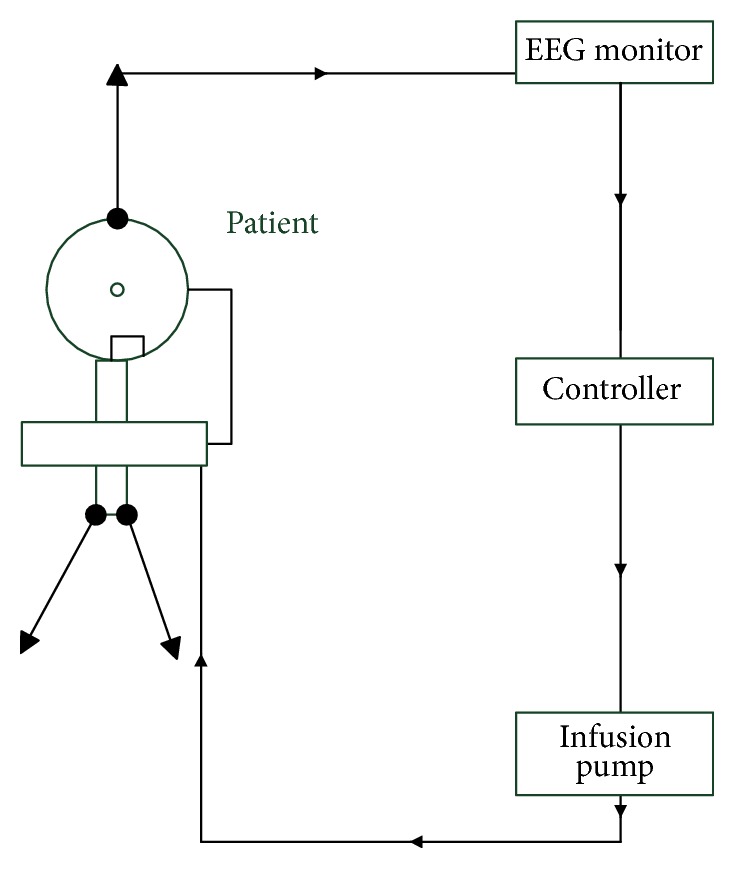
Closed loop drug infusion in anesthesia.

In Sections 2 and 3, we have analyzed anesthesia administration and presented patient modeling.  Figure 4 presents the closed loop anesthesia system with BIS signal as feedback and drug infusion through an infusion pump (IP) [62].

### 4.1. Target Controlled Infusion

Target controlled infusion (TCI) is an open loop control system. The reference point is set by anesthesiologist and controller maintains the reference level. But such controller is not immune to uncertainties and has no robustness. If there is a change in DOH levels during skin incision, the controller has no ability to adjust them and to attain the desired level [57, 58, 63]. TCI administers the optimized level of drug dosage. TCI pump uses algorithm based on pharmacological data obtained from healthy volunteers. Such schemes may be less accurate when applied during extreme situation of surgery including considerable loss of blood [27].  Table 3 presents Propofol concentration level required at different compartment of the patients including blood and brain [24].

### 4.2. Proportional Integral Derivative Control

Proportional integral derivative (PID) controller is a classical control technique that is widely used in chemical process industry. Its importance lies in its fast transient response and greater ability to reduce the steady-state error. Closed loop control of anesthesia was introduced by Dong in 2003 [37], where he derived the compartmental model of the patient and linearized it by using linear regression. He applied PID control technique on hypothetical patient and achieved the desired hypnosis level. However, by linearizing the nonlinear sigmoid model, big amount of data can be lost and accurate results cannot be obtained [37].

Soltész worked on the same procedure to control hypnotic and analgesic component of anesthesia [9]. He developed a closed loop system using hypnotic drug Propofol and analgesic Remifentanil. Soltész used the PID control technique having adaptive behavior in which the controller tracked the desired hypnosis level. For Remifentanil, he used the P controller. Patient model was derived from the clinical data. The drug infusion was controlled during the maintenance and induction phases of anesthesia [9]. The limitation of TCI is due to the interpatient variability. Interpatient variability is the variation in different dynamics among the different patients including age, height, and weight. These variables are changing from patient to patient and each patient shows different response to the drug infusion. Closed loop control system has the ability to reduce the effect of interpatient variability [6]. Closed loop control reduces the workload of anesthesiologist. Practical experiments were carried out on 47 validated modules which achieved interpatient variability. Such study has been observed for 6–16-year-old children. To measure the depth of hypnosis, neurosense monitor was used in [18]. The controlled design for Propofol and Remifentanil is multi-input but Remifentanil metabolizes faster than Propofol [38]. A robustly tuned PID controller performs well to bring the hypnosis level to a desired value. Such controller is based on identified patient parameters and possesses greater ability for disturbance rejection, such as surgical stimuli. This was validated on a dataset of 44 patients from clinical trials [39]. Implementation of PID is simple but its tuning is quite difficult. Moreover, the control performance of PID is limited which leads to instability of the closed loop system [34, 35, 40].

### 4.3. Sliding Mode Control

Sliding mode control (SMC) is one of the most effective control techniques to design robust controller for higher-order nonlinear systems in uncertain environments. The main functionality of SMC is moving the state trajectory of a plant towards user defined surface. The major benefits of SMC are low sensitivity to plant disturbances and uncertainties [41].

Propofol is an intravenously administered anesthetic agent that is commonly used for induction and maintenance of anesthesia. SMC controller is designed based on a set of state equations derived from PK and PD models that describe different behavior and responses of the patient to Propofol infusion. Inducing and maintaining of anesthesia in feedback control system depend on the Wavelet Anesthetic Value for central nervous system (WAV_CNS_). Initially, the Higher-Order Sliding Mode Controller (HOSMC) is used for insulin infusion and glucose monitoring in type 1 diabetic patients. Later on, this technique is applied for anesthesia using Propofol infusion in a general surgery. The major advantage of HOSMC is its less sensitivity towards patient parameters. Second advantage of HOSMC is its robustness as compared to other control techniques such as predicative control in the presence of bleeding which is a surgical stimulus during a surgical procedure [41].

### 4.4. Adaptive Fuzzy Sliding Mode Control

A major limitation of SMC is the chattering phenomena. A robust control strategy is needed for smooth execution of surgical procedure, while handling the interpatient variability and external disturbances. The adaptive SMC scheme is used for controlling depth of anesthesia. To address the chattering phenomena and uncertainties, adaptive fuzzy SMC systems are applied with neural control. Adaptive fuzzy SMC tracks the system trajectory towards sliding surface, while neural control is used as a secondary controller for the cases when system states move towards the boundary layer. Experiments were carried out on 8 patients and their simulation results establish that the proposed approach gives reliable performance. It shows several advantages over others, such as lesser settling time and generating smooth input signal, thus avoiding the risk of overdosing and underdosing [42]. Fuzzy logic control is based on fuzzy set of operations and functions like Gaussian, trapezoidal, and triangular. The controller was applied and assessed for population of 1000 different intraoperative patients. To develop fuzzy controller, it is necessary to identify the input variables for classification of patients' compartments including the model of effect site compartment [43, 44]. Fuzzy logic system for logical operation was introduced by Zadeh in 1965 [45]. The major limitation of this scheme is the fidelity of patient variability model termed as interpatient variability [46].

### 4.5. Model Predicative Control

A major problem with fixed parameter PID controller is its vast deviation from the actual output in the form of a steady-state error. Adaptive control strategies easily overcome this inherent limitation of a PID controller. Nonlinear adaptive control has been used for controlling BIS level [15].

Model predicative control (MPC) algorithm is an optimal control algorithm which is also used for Propofol anesthesia administration. It has a number of features, such as BIS tracking, noise rejection, and disturbance handling capability. MPC is also known as moving horizon control algorithm. It has a number of applications in process industry and greater capability of handling critical applications like anesthesia control or glucose control. Performance of linear model predicative controller (LMPC) is comparable to PID considering the time delay introduced by BIS monitor during anesthesia control. LMPC has been found to be robust against interpatient and intrapatient variability and towards noise handling and disturbance compensation [47]. Intrapatient variability means different dynamics among the same patient including blood pressure variation, changes in hypnosis due to blood loss, awareness of the patient during surgery, trauma situation, and unexpected prolongation of procedure.

The new variant of predicative control is the robust predicative control algorithm which has been applied in anesthesia administration [48]. Here, a single input (Propofol), a single output (BIS), and an output variable model of patient has been used for predication as well as simulation. A group of 12 patient models were studied, analyzed, and designed using robust predicative controller which ensured that interpatient variability is handled properly. The nonlinearity of the patient model causes nonlinear gain during a controller design which also varies with respect to interpatient variability. LMPC ensures stability and Propofol administration accurately, resulting in keeping the hypnotic level in a desirable range. The performance of robust predicative controller has been clinically accepted [48].

### 4.6. Backstepping Control

It is a recursive control algorithm used for stabilizing nonlinear dynamical system. Here, the high-order system is reduced to lower-order system [49]. This method shows that output tracks the desired reference level of hypnosis during surgery. The most vital performance of backstepping control algorithm is to handle useful nonlinearities in spite of cancelling them [50].

### 4.7. Internal Model Control

Internal model control (IMC) is the basic control technique using BIS signal as feedback for monitoring the depth of anesthesia. The main significance of IMC is its adjustable parameter in the structure. The performance parameter like settling time can be easily adjusted in closed loop implementation of IMC [51]. If the abrupt change in hypnosis level occurs, then it is difficult to judge whether it is the patient dynamic variation or BIS fault. Fault-tolerant internal model control system is a better solution for the closed loop anesthesia system. It can identify fault or dynamic variation of the patient [52].

### 4.8. Adaptive Control

In the schemes discussed so far, the major limitations are to handle the interpatient and intrapatient variability. Regression model is used for the prediction of patient response and to provide the adequate dosage level to keep patient hypnosis level in the desirable range. Predicative adaptive controller has ability to continuously monitor the patients' responses and compute the drug level in order to maintain the specified BIS target [64]. Adaptive neural networks are introduced to improve the Reinforcement Learning (RL) for administering Propofol to regulate hypnosis. The proposed controller is tested on in silico patients and compared to other linear control schemes. It is observed that it outperformed other techniques [53]. RL is an intelligent control strategy that has shown clinically acceptable BIS-guided DOH level in in silico as well as real patients [54, 55].

### 4.9. *H*_*∞*_ Control

The depth of anesthesia (DoA) model can be linearized around operating point using Wiener nonlinear structure. *H*_*∞*_ design method is based on continuous linear controller to ensure robust stability and compensate uncertainty in patient dynamics during surgical procedures [36, 56].

### 4.10. Software Platform for Anesthesia Administration

Distributed software platform and parallel computer architecture are used for control of anesthesia administration. Such a system is a prototype base intended to help the development for simulation and test of new algorithm for anesthesia process. The software platform system consists of two computers allocated for anesthesia control and process supervision. The first computer receives physiological data from sensors, simulating control algorithm and commanding the actuators to provide adequate infusion rate. The main function of the second computer is to supervise and configure the control operation [59, 60]. The application of TANGO framework increased the reliability of the interconnection between several software modules and distributed units. This network can be easily adapted to a more complex control problem complying with real-time scenario [59, 61].

### 4.11. Hardware Platform for Anesthesia Administration

Ethicon Endo-Surgery Inc. introduced the first Computer-Assisted Personalized Sedation (CAPS) system named SEDASYS which is used for automating the administration of anesthesia to relatively healthy patients during colonoscopies. It also measures the oxygen saturation, blood pressure, capnometry, respiration, electrocardiography, patient responsiveness, and heart rate of the sedated patients. It is a safe source to improve care and reduces costs of colonoscopies [65]. It has been approved for use in Canada, Australia, and the EU. However, the American Society of Anesthesiologists issued several guidelines for the use of SEDASYS in 2014 but did not endorse the system [66]. Researchers at McGill University in Canada also developed an automated monitoring and drug delivery device named McSleepy [67].

## 5. Discussion and Assessment of Closed Loop Anesthesia

The main objective of anesthesia administration is to execute surgical procedure safely. The anesthesiologist is responsible for maintaining all phases of anesthesia including induction, maintenance, and emergence phases. The surgical procedure is executed during maintenance phase. Anesthetics are administered during induction phase of anesthesia. Moreover, the emergence phase is initiated after skin closure. In manual administration of anesthesia, the patient is given analgesic and hypnotics as well as areflexia. As the surgical procedure gets prolonged, the anesthesiologist administers these drugs in appropriate fashion. Serious medical complications can develop due to underdosing and overdosing of these drugs. These lead to the motivation of automation in anesthesia. Compartment model of human body is derived based on PK and PD. It divides the human body into four compartments, blood, muscle, fat, and brain. The BIS monitor extracts information from EEG and gives at its output the hypnosis level values which are fed back to the controller, hence forming a closed loop system. Different control algorithms including linear, nonlinear, robust, adaptive, and artificial intelligence algorithms show different results for automation in anesthesia. Linear controllers like PID are classical control schemes applied in process industry. PID delivers fast transient response and less steady-state error but cannot handle nonlinearity and disturbance like incision in closed loop anesthesia system. SMC can easily handle the nonlinearity and chattering in hypnosis level. Sometimes loss of blood occurs during surgical procedure which can affect the hypnosis level and it may go to an undesirable level. Observer based estimation of hypnosis level can prove helpful to handle undesirable circumstances in hypnosis level by identifying the issue which might have occurred in BIS or patient dynamics. Oscillation in hypnosis is improved with application of adaptive fuzzy SMC. Predicative control technique performed well in observing state estimation and disturbance rejection. State estimation proved helpful in observing the drug flow in different body parts like muscle, fat, and bone. Blood acts as carrier for the drug to different parts of the body. Metabolism of drug within human body depends on age, height, and weight of the patients. The main challenges in automation of anesthesia are handling of interpatient and intrapatient variability. Sudden faults in infusion pump, BIS monitor, and EEG monitor are some of the challenges for modern research on automation in closed loop systems. The proposed SMC based algorithm achieves hypnosis level between 40 and 60. To design SMC, the sliding surface is given by the following equations:

(15) $$\begin{array}{c} \sigma =a_{1}m_{1}+a_{2}m_{2}+a_{3}m_{3}+a_{4}m_{e} \end{array}$$

or

(16) $$\begin{array}{c} \dot{\sigma }=a_{1}{\dot{m}}_{1}+a_{2}{\dot{m}}_{2}+a_{3}{\dot{m}}_{3}+a_{4}{\dot{m}}_{e}, \end{array}$$

where *a*_1_, *a*_2_, *a*_3_, *a*_4_ are tuning parameters of the controller. With *a*_1_ = 1, values of other parameters are chosen in a way that *σ* becomes Hurwitz monic polynomial. Putting the value of ${\dot{m}}_{1},{\dot{m}}_{2},{\dot{m}}_{3},{\dot{m}}_{e}$ from (1)–(4), respectively,

(17) σ˙=a1−k10−k12−k13m1t+k21m2t+k31m3t+ut+a2k12m1t−k21m2t+a3k13m1t−k31m3t+a4k1em1t−ke0met.

The overall control law (*u*) consists of equivalent control (*u*_eq_) and discontinuous control (*u*_disc_) as

(18) $$\begin{array}{c} u=u_{eq}+u_{disc}. \end{array}$$

The equivalent control forces the system dynamics to move to the sliding surface and depends on the states of the system and state parameters. It makes the derivative of sliding manifold equal to zero and can be computed by putting $\dot{\sigma }=0$ along the system dynamics (17). Thus,

(19) ueq=−−k10−k12−k13m1t+k21m2t+k31m3t−a2k12m1t−k21m2t−a3k13m1t−k31m3t−a4k1em1t−ke0met.

Presence of disturbances or uncertainties may result in *σ* ≠ 0. Discontinuous control handles such disturbances and depending on the gain and signum function exhibits switching behavior. Thus,

(20) $$\begin{array}{c} u_{disc}=-k\;sign\left(\;\sigma \;\right). \end{array}$$

**Figure 5:**
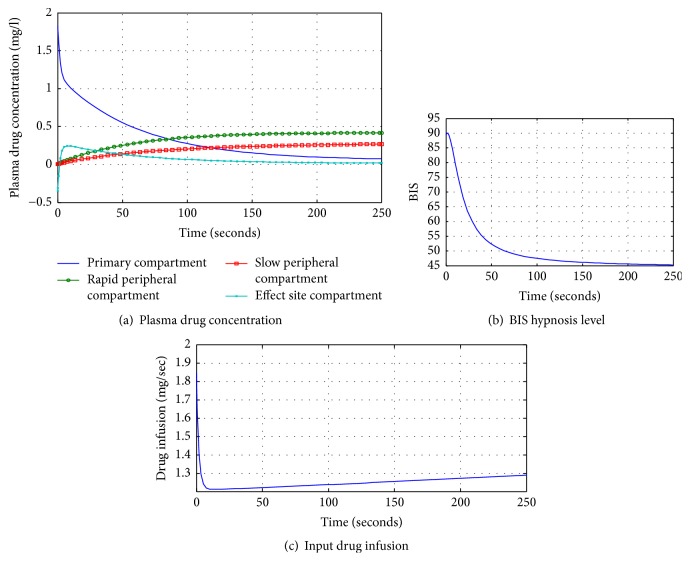


Figure 5(a) represents the plasma drug concentration in different compartments. Initially, the drug is administered into primary compartment. The drug is distributed from the primary compartment in an exponential fashion. As the drug level decreases in the primary compartment, it leads to frequent increases in rapid peripheral compartment and then gradual increases in slow peripheral compartment. Moreover, the drug metabolism rate depends on patient dynamics like age, height, and weight.  Figure 5(b) shows the hypnosis level of the in silico patient during surgical procedure. In first 100 seconds, it achieves the maintenance phase that leads to initiation of surgical procedure. After 100 seconds, hypnosis level is attained between 40 and 60 for smooth execution of surgical procedure.  Figure 5(c) represents the drug infusion indicating the maximum level at start followed by slow decay achieving a stable position.

Table 4 shows the tabular analysis and assessment of different control algorithms. Different anesthetic drugs are analyzed in terms of their merits and demerits. The administered drugs including painkillers also affect the digestive system of the patients. Nitrous oxide is a volatile drug which is used as an inhaler and acts as a hypnotic agent. Linear and nonlinear control algorithms are analyzed and compared in terms of transient response, overshoot, and steady-state error in Table 4. Linear control schemes exhibit fast transient response but cannot handle uncertainties and disturbances as compared to nonlinear schemes. Adaptive controller exhibits adaptive behavior in uncertain environments, yet it provides optimum results. The proposed SMC scheme is a nonlinear controller which outperforms linear control schemes. It is clear from the above-mentioned comparison between automation and manual methods that automation in anesthesia can easily overcome problems caused by the latter administration of anesthesia.

## 6. Conclusion

This review article presents and compares different aspects of control algorithms useful in handling prominent complications occurring in closed loop anesthesia infusion system. General surgical procedures executed in well-equipped operation theatre are inclining towards automated drug delivery systems replacing manual anesthetics infusion. Recent research work on automated drug infusion signifies the importance of nonlinear and robust control strategies as compared to linear control schemes because they can cope well with nonlinearities and uncertainties occurring in natural phenomenon. The proposed control strategy based on SMC shows optimum results for smooth execution of general surgical procedures. Interpatient and intrapatient variability handling is the key challenge in modeling and design of automated anesthesia drug delivery systems.

## Figures and Tables

**Table 1:** Required effect site concentration for commonly used closed loop anesthesia.

Drug	Effect	Required effect site concentration
Propofol	Sedation	2-3 *μ*g·ml^−1^
	Anesthesia	4–6 *μ*g·ml^−1^

Remifentanil	Laryngoscopy	2-3 ng·ml^−1^
	Analgesia for superficial surgery	3-4 ng·ml^−1^
	Analgesia for laparotomy	6–8 ng·ml^−1^
	Analgesia for cardiac surgery	10–12 ng·ml^−1^

Alfentanil	Analgesia for major surgery	75–100 ng·ml^−1^
	Analgesia for cardiac surgery	150–220 ng·ml^−1^

Sufentanil	Analgesia for major surgery	0.1–0.4 ng·ml^−1^
	Analgesia for cardiac surgery	0.6–1.0 ng·ml^−1^

**Table 2:** Nomenclature of clinical parameters.

Symbol	Unit	Name
*u*(*t*)	mg·sec^−1^	Infusion rate
*k*_10_	sec^−1^	Elimination rate constant
*m*_1_	mg	Amount of drug in primary compartment
*m*_2_	mg	Amount of drug in rapid peripheral compartment
*m*_3_	mg	Amount of drug in slow peripheral compartment
*m*_*e*_	mg	Flow of hypnotic agent in effect site
*k*_1*e*_	sec^−1^	Rate constant at effect site
*k*_*e*0_	sec^−1^	Elimination rate constant at effect site

**Table 3:** Targeted control infusion (5 *μ*g·ml^−1^) based on calculated blood concentration using Paedfusor PK model.

	Blood concentration targeting	Effect site concentration targeting
Loading dose	1.7 mg·kg^−1^	5.7 mg·kg^−1^
Maximum blood target reached	5 mcg·kg^−1^	12 mcg·kg^−1^
Total Propofol infused after 60 min	23.2 mg·kg^−1^	23.3 mg·kg^−1^
Time to achieve effect site target of 5 mcg·ml^−1^	17.5 min	4.5 min

**Table 4:** Comparative analysis of various control mechanisms and clinical tools employed in anesthesia.

Terminology	Action	Merits	Demerits	References
Anesthesia	Lack of sense	Applied in surgical procedure	Effect digestive system, vomiting, and so forth	[1–4, 6, 8–11, 15, 18–21, 23, 25, 35, 36]

Propofol	Anesthetic agent	Fast metabolic action, less side effects, being easily recoverable	No	[1, 2, 4–7, 9, 11, 13, 16, 18–23, 28]

Remifentanil	Analgesic, painkiller	Less side effect, providing relief from pain, no postoperative effect	Excessive amount affects the stomach	[17–19, 24]

Nitrous oxide	Inhale volatile drugs	Used as painkiller	Not purely hypnotic	[1, 16, 23]

PID controller	Linear control technique	Fast transient response, showing adaptive behavior	Linearizing the data leads to loss of information. Cannot cope with uncertainties	[9, 18, 30–32, 34, 35, 37–40]

Sliding mode control	Nonlinear control scheme	Handling uncertainties like skin incision, less steady error up to 5%	Chattering is observed in hypnosis level	[40, 41]

Adaptive fuzzy SMC	Robust control scheme	Handling chattering in maintenance phase of anesthesia	Steady-state error still exists	[42–46]

Model predicative control	Optimal control strategy	Noise rejection of Intense care equipment, hypnosis level tracking	Settling time of achieving hypnosis can further be improved; steady-state error is 5%	[15, 47, 48]

Robust predicative control	Robust control scheme	Handling interpatient and intrapatient variability	No serious issues. Result is clinically accepted	[47]

Backstepping control	Nonlinear control algorithm	Fast transient response	Steady-state error exists	[49, 50]

Internal model control	Robust control scheme	Handling dynamics in hypnosis level	Complication in handling uncertainty	[51, 52]

Adaptive control	Used in adaptive model	Handling interpatient variability	Complex mathematics involved	[36, 47, 53–55]

*H*_*∞*_ control	Based on linear model	Handling uncertainly	Data lost in linearizing model	[36, 56]

TCI	Open loop system	Being easily applicable	Unable to compensate disturbances	[1, 5, 27, 57, 58]

BIS	Display cortical activity of brain	Extracting the inform of DOH from EEG easily	Unable to compensate noise of other equipment in ICU	[5, 8–10, 16, 18, 19, 22, 25, 26, 34]

TANGO	Software platform	Supervisory network for sensing as well as control purpose	Not viable for compensating interpatient variability	[59–61]
